# Annotations

**Published:** 1902-08-23

**Authors:** 


### Annotations.

### The Birmingham Guardians and Labour Colonies.

At a recent meeting of the Birmingham Guardians a suggestion was made to send tramps to labour colonies, but it met with but scant success. The chairman, Mr. H. J. Sayer, considered the scheme utopian. He was sure that the ratepayers would not consent, and he did not seem to think it likely that the tramps would consent either. Tramps, he said, would not work. They had no ambition, no family affection, nothing on which to touch with a view to elevating them. Another member, noting that the proposer of the motion had mentioned that 10,720 tramps had passed through the casual wards of the Birmingham Union in six months, declared that to provide for these would want not an agricultural colony but an agricultural county. Mr. Robinson, who supported the scheme, pleaded that these colonies, even though they were not financially self-supporting, were successful in raising and reclaiming many of their inhabitants, and expressed his belief that many persons now on tramp would be willing to do honest work if they could get it. Even without endorsing this view of the average tramp, one may think that an agricultural colony might be a wise experiment in poor-law management. The honest men would get honest work, and might finally, when trained and tested, be drafted to some colony oversea where they might have a better chance of earning a decent living. And the others—the members of the "Society of the Sons of Everlasting Rest" as one guardian sarcastically called them, when they found that an appeal for assistance from the Birmingham guardians resulted in their being sent for a considerable time to a colony where they would have to work every day and all day long, would rather go elsewhere than try. In a recent number of the *Strand Magazine* there was an illustrated article dealing with the language of tramps, and showing some of the signs by which one member of that fraternity informs a succeeding one what reception he may expect at different houses. A circle chalked on door or wall means "No use to call here." The establishment of an agricultural colony might mean the drawing of a circle on the door of the Birmingham Union, and the majority of the 10,720 whose maintenance Mr. Watson feared, would try some other place.

### Compulsory Notification of Consumption.

A very useful discussion on the prevention of tuberculosis took place at the Manchester meeting of the British Medical Association, in which some of the speakers insisted strongly upon the necessity of securing compulsory notification of consumption. We have again and again pointed out the difficulties which stand in the way of including tuberculosis in the schedule of the Notification Act, and notwithstanding all the hard things which have been said against the Local Government Board, we feel sure that the position they have taken up in the matter is the proper one in the present stage of the problem. That it is so is shown by the altered temper in which the question is now approached by the advocates of notification. Dr. Robertson, himself a strong advocate of notification, very properly said that different infectious diseases ought to be dealt with under different bye-laws or regulations. While, then, not asking for powers to interfere with any person's freedom, as that term is usually applied, he held strongly that the time had arrived when we might demand compulsory notification of tuberculosis with the object of insisting upon the expectoration being properly dealt with and the necessary disinfection being effectually done. This appears, on the face of it, a reasonable proposition, and is, of course, a very different thing from scheduling tuberculosis as a dangerous infectious disease; but even such powers as would be given under this arrangement would require to be applied with much care and judgment—more judgment, in fact, than can be properly credited to all sanitary authorities. The question of the wage-earner remains a standing difficulty. It seems to us idle to pretend that if consumptive cases are to be properly "followed up" their opportunities for obtaining employment will not be interfered with, and we are quite clear that Dr. Robertson's first proposal of "a special notification enactment" leads very logically to his last, namely, "a special enactment dealing with the provision of places for the treatment of consumptives." Will the "authorities," *i.e.*, the ratepayers, who demand the one provide the other?

### Heavy Fees and Wealthy Patients.

A surgeon had a patient who was a wealthy man. Now this patient had a not very extraordinary illness, and for its treatment the doctor did an operation, which again was not of a very extraordinary character. It was well and carefully done, however, and the patient making a good recovery, was by its means restored once more to the enjoyment of his great wealth. He evidently had his reward, and that a big one; so the doctor thought that he also deserved something out of the common way, and therefore demanded a heavy fee. The patient, however, did not see the reasonableness of the suggestion. Like many a rich man he knew the value of money and he declined to pay, offering however a very much smaller amount—which was accepted! The question is now being put, as a sort of problem in medical ethics, What ought the doctor to have done? Clearly a medical man has a right to charge what he likes for his services, and it is a recognised custom to charge, to some extent, according to the position of the patient; so that he had something on his side. But if he wishes to demand fees widely differing from those accepted by others, and especially if in a given case, as in that of the wealthy patient, he intends to demand a far higher fee than he himself usually accepts for the same service, he surely ought to say so beforehand, and leave even the poor millionaire a choice as to whether to pay the greater fee or accept the service of the lesser doctor. To charge a heavy fee and then to climb down when admittedly there was no question as to the means of the patient, was to say the least of it ignominous, and strongly suggestive of an attempt at extortion. Better to have demanded all or none. Better still to have made an ordinary business arrangement beforehand.

